# Maximum Entropy Expectation-Maximization Algorithm for Fitting Latent-Variable Graphical Models to Multivariate Time Series

**DOI:** 10.3390/e20010076

**Published:** 2018-01-19

**Authors:** Saïd Maanan, Bogdan Dumitrescu, Ciprian Doru Giurcăneanu

**Affiliations:** 1 Department of Statistics, University of Auckland, Auckland 1142, New Zealand; 2 Department of Automatic Control and Computers, University Politehnica of Bucharest, 060042 Bucharest, Romania

**Keywords:** maximum entropy, Expectation-Maximization, graphical models, autoregressive model, latent variables, information theoretic criteria, time series

## Abstract

This work is focused on latent-variable graphical models for multivariate time series. We show how an algorithm which was originally used for finding zeros in the inverse of the covariance matrix can be generalized such that to identify the sparsity pattern of the inverse of spectral density matrix. When applied to a given time series, the algorithm produces a set of candidate models. Various information theoretic (IT) criteria are employed for deciding the winner. A novel IT criterion, which is tailored to our model selection problem, is introduced. Some options for reducing the computational burden are proposed and tested via numerical examples. We conduct an empirical study in which the algorithm is compared with the state-of-the-art. The results are good, and the major advantage is that the subjective choices made by the user are less important than in the case of other methods.

## 1. Introduction

Graphical models are instrumental in the analysis of multivariate data. Originally, these models have been employed for independently sampled data, but their use has been extended to multivariate, stationary time series [1,2], which triggered their popularity in statistics, machine learning, signal processing and neuroinformatics.

For better understanding the significance of graphical models, let x be a random vector having a Gaussian distribution with zero-mean and positive definite covariance matrix Γ. A graph G=(V,E) can be assigned to x in order to visualize the conditional independence between its components. The symbol *V* denotes the vertices of *G*, while *E* is the set of its edges. There are no loops from a vertex to itself, nor multiple edges between two vertices. Hence, *E* is a subset of $\left\{(a,b)\in V\times V:a\neq b\right\}$. Each vertex of the graph is assigned to an entry of x. We conventionally draw an edge between two vertices *a* and *b* if the random variables $x_{a}$ and $x_{b}$ are *not* conditionally independent, given all other components of x. The description above follows the main definitions from [3] and assumes that the graph *G* is *undirected*. Proposition 1 from the same reference provides a set of equivalent conditions for conditional independence. The most interesting one claims that $x_{a}$ and $x_{b}$ are conditionally independent if and only if the entry (a,b) of $\Gamma^{-1}$ is zero. This shows that the missing edges of *G* correspond to zero-entries in the inverse of the covariance matrix, which is called the concentration matrix.

There is an impressive amount of literature on graphical models. In this work, we focus on a generalization of this problem to time series. The main difference between the *static* case and the *dynamic* case is that the former relies on the sparsity pattern of the concentration matrix, whereas the latter is looking for zeros in the inverse of the spectral density matrix. One of the main difficulties stems from the fact that the methods developed in the static case cannot be applied straightforwardly to time series.

Parametric as well as non-parametric methods have been already proposed in the previous literature dedicated to graphical models for time series. Some of the recently introduced estimation methods are based on convex optimization. We briefly discuss below the most important algorithms which belong to this class.

- Reference [4] extends the static case by allowing the presence of latent variables. The key point of their approach is to express the manifest concentration matrix as the sum of a sparse matrix and a low-rank matrix. Additionally, they provide conditions for the decomposition to be unique, in order to guarantee the identifiability. The two matrices are estimated by minimizing a penalized likelihood function, where the penalty involves both the $\ell_{1}$-norm and the nuclear norm. Interestingly enough, the authors of the discussion paper [5] pointed out that an alternative solution, which relies on the Expectation-Maximization algorithm, can be easily obtained.
- In the dynamic case, reference [6] has an important contribution which consists in showing that the graphical models for multivariate autoregressive processes can be estimated by solving a convex optimization problem which follows from the application of the Maximum Entropy principle. This paved the way for the development of efficient algorithms dedicated to topology selection in graphical models of autoregressive processes [7,8] and autoregressive moving average processes [9].
- A happy marriage between the approach from [4] and the use of Maximum Entropy led to the solution proposed in [10] for the identification of graphical models of autoregressive processes with latent variables. Similar to [4], the estimation is done by minimizing a cost function whose penalty term is given by a linear combination of the $\ell_{1}$-norm and the nuclear norm. The two coefficients of this linear combination are chosen by the user and they have a strong influence on the estimated model. The method introduced in [10] performs the estimation for various pairs of coefficients which yield a set of candidate models; the winner is decided by using a *score function*.

According to the best of our knowledge, there is no other work that extends the estimation method from [5] to the case of latent-variable autoregressive models. The main contribution of this paper is to propose an algorithm of this type, which combines the strengths of Expectation-Maximization and convex optimization. The key point for achieving this goal is to apply the Maximum Entropy principle.

The rest of the paper is organized as follows. In the next section, we introduce the notation and present the method from [10]. Section 3 outlines the newly proposed algorithm. The outcome of the algorithm is a set of models from which we choose the best one by employing information theoretic (IT) criteria. Section 4 is focused on the description of these criteria: We discuss the selection rules from the previous literature and propose a novel criterion. The experimental results are reported in Section 5. Section 6 concludes the paper.

## 2. Preliminaries and Previous Work

Let $x_{1},\ldots ,x_{T}$ be a κ-dimensional (κ>1) time series generated by a stationary and stable VAR process of order *p*. We assume that the spacing of observation times is constant and $x_{t}={\left[x_{1t},\ldots ,x_{\kappa t}\right]}^{⊤}$. The symbol ${\left(\cdot \right)}^{⊤}$ denotes transposition. The difference equation of the process is

(1) $$x_{t}=A_{1}x_{t-1}+\ldots +A_{p}x_{t-p}+\epsilon_{t},\;t=\bar{1,T},$$

where $A_{1},\ldots ,A_{p}$ are matrix coefficients of size κ×κ and $\epsilon_{t}$ is a sequence of independently and identically distributed random κ-vectors. We assume that the vectors ${\left\{\epsilon_{t}\right\}}_{t=1}^{T}$ are drawn from a κ-variate Gaussian distribution with zero mean vector and covariance matrix Σ≻0. Additionally, the vectors ${\left\{x_{t}\right\}}_{t=1-p}^{0}$ are assumed to be constant.

The conditional independence relations between the variables in $x_{t}$ are provided by the inverse of the spectral density matrix (ISDM) of the VAR-process $\left\{x_{t}\right\}$. The ISDM has the expression

(2) $$\Phi^{-1}\left(\omega \right)=A^{H}\left(\omega \right)\Sigma^{-1}A\left(\omega \right)=\sum_{i=-p}^{p}Q_{i}e^{-j\omega i},$$

where $j=\sqrt{-1}$ and ${\left(\cdot \right)}^{H}$ is the operator for conjugate transpose. We define $A_{0}=-I$, where I stands for the identity matrix of appropriate size, and $A\left(\omega \right)=-\sum_{i=0}^{p}A_{i}e^{-j\omega i}$. For i≥0, we have that $Q_{i}=\sum_{k=0}^{p-i}A_{k}^{⊤}\Sigma^{-1}A_{k+i}$ and $Q_{-i}=Q_{i}^{⊤}$. The sparse structure of the ISDM contains conditional dependence relations between the variables of $x_{t}$, i.e., two variables $x_{a}$ and $x_{b}$ are independent, conditional on the other variables, if and only if [1,11]

(3) $${\left[\Phi^{-1}\left(\omega \right)\right]}_{ab}=0,\;\forall \omega \in \left(-\pi ,\pi \right].$$

In the graph corresponding to the ISDM $\Phi^{-1}\left(\omega \right)$, the nodes stand for the variables of the model, and the edges stand for conditional dependence, i.e., there is no edge between conditionally independent variables.

In a latent-variable graphical model it is assumed that κ=K+r, where K variables are accessible to observation (they are called manifest variables) and r variables are latent, i.e., not accessible to observation, but playing a significant role in the conditional independence pattern of the overall model. The existence of latent variables in a model can be described in terms of the ISDM by the block decomposition

(4) $$\Phi \left(\omega \right)=\left[\begin{array}{cc} \Phi_{m}\left(\omega \right) & \Phi_{\ell m}^{⊤}\left(-\omega \right)\\ \Phi_{\ell m}\left(\omega \right) & \Phi_{\ell }\left(\omega \right) \end{array}\right],\;\Phi^{-1}\left(\omega \right)=\left[\begin{array}{cc} \Upsilon_{m}\left(\omega \right) & \Upsilon_{\ell m}^{⊤}\left(-\omega \right)\\ \Upsilon_{\ell m}\left(\omega \right) & \Upsilon_{\ell }\left(\omega \right) \end{array}\right],$$

where $\Phi_{m}\left(\omega \right)$ and $\Phi_{\ell }\left(\omega \right)$ are the manifest and latent components of the spectral density matrix, respectively. Using the Schur complement, the ISDM of the manifest component has the form [10] (Equation (21)):

(5) $$\Phi_{m}^{-1}\left(\omega \right)=\Upsilon_{m}\left(\omega \right)-\Upsilon_{\ell m}^{⊤}\left(-\omega \right)\Upsilon_{\ell }^{-1}\left(\omega \right)\Upsilon_{\ell m}\left(\omega \right).$$

When building latent variable graphical models, we assume that r≪K, i.e., few latent variables are sufficient to characterize the conditional dependence structure of the model. The previous formula can therefore be written

(6) $$\Phi_{m}^{-1}\left(\omega \right)=S\left(\omega \right)-\Lambda \left(\omega \right),$$

where S(ω) is sparse, and Λ(ω) has (constant) low-rank almost everywhere in (−π,π]. Furthermore, we can write [12] (Equation (4)):

(7) $$\begin{array}{c} S\left(\omega \right)-\Lambda \left(\omega \right)=\Delta \left(\omega \right)X\Delta {\left(\omega \right)}^{H}, \end{array}$$

(8) $$\begin{array}{c} \Lambda \left(\omega \right)=\Delta \left(\omega \right)L\Delta {\left(\omega \right)}^{H}, \end{array}$$

where $\Delta \left(\omega \right)=\left[I,e^{j\omega }I,\ldots ,e^{j\omega p}I\right]$ is a shift matrix, and X and L are $K\left(p+1\right)\times K\left(p+1\right)$ positive semidefinite matrices. We split all such matrices in K×K blocks, e.g.,

(9) $$L=\left[\begin{array}{ccc} L_{00} & \ldots & L_{0p}\\ \vdots & \ddots & \vdots \\ L_{0p}^{⊤} & \ldots & L_{pp} \end{array}\right].$$

The block trace operator for such a matrix is D(·), defined by

(10) $$D_{i}\left(L\right)=\sum_{h=0}^{p-i}L_{h,h+i},\;i=\bar{0,p}.$$

For negative indices, the relation $D_{-i}\left(L\right)=D_{i}{\left(L\right)}^{⊤}$ holds. Note that (8) can be rewritten as

$$\Lambda \left(\omega \right)=\sum_{i=-p}^{p}D_{i}\left(L\right)e^{j\omega i}.$$

The first p+1 sample covariances of the VAR process are [13]:

(11) $${\hat{C}}_{i}=\frac{1}{T}\sum_{t=1}^{T-i}x_{t+i}x_{t}^{⊤},\;i=\bar{0,p}.$$

However, only the upper left K×K blocks corresponding to the manifest variables can be computed from data; they are denoted ${\hat{R}}_{i}$. With $\hat{R}=\left[{\hat{R}}_{0}\ldots {\hat{R}}_{p}\right]$, we build the block Toeplitz matrix

(12) $$T\left(\hat{R}\right)=\left[\begin{array}{ccc} {\hat{R}}_{0} & \ldots & {\hat{R}}_{p}\\ \vdots & \ddots & \vdots \\ {\hat{R}}_{p}^{⊤} & \ldots & {\hat{R}}_{0} \end{array}\right].$$

It was proposed in [10] to estimate the matrices X and L by solving the optimization problem

(13) $$\begin{array}{c} \begin{array}{cc} \min_{X,L} & \mathrm{tr}\left(T\left(\hat{R}\right)X\right)-\log\det X_{00}+\lambda \gamma f\left(X+L\right)+\lambda \mathrm{tr}\left(L\right)\\ s.t. & X⪰0\;L⪰0, \end{array} \end{array}$$

where tr(·) is the trace operator, log(·) denotes the natural logarithm and det(·) stands for the determinant. Minimizing tr(L) induces low rank in L and λ,γ>0 are trade-off constants. The function f(·) is a group sparsity promoter whose expression is given by

(14) $$f\left(Z\right)=\sum_{a=1}^{K}\sum_{b=1}^{a-1}\max_{i=\bar{0,p}}\left|D_{i}\left(Z\right)\left(a,b\right)\right|.$$

Note that $D_{i}\left(X+L\right)\left(a,b\right)$ is the *i*-th degree coefficient of the polynomial that occupies the (a,b) position in the matrix polynomial $\Phi_{m}^{-1}\left(\omega \right)$. Sparsity is encouraged by minimizing the $\ell_{1}$-norm of the vector formed by the coefficients that are maximum for each position (a,b).

## 3. New Algorithm

The obvious advantage of the optimization problem (13) is its convexity, which allows the safe computation of the solution. However, a possible drawback is the presence of two parameters, λ and γ, whose values should be chosen. A way to eliminate one of the parameters is to assume that the number r of latent variables is known. At least for parsimony reasons, it is natural to suppose that r is very small. Since a latent variable influences all manifest variables in the ISDM (5), there cannot be too many independent latent variables. Therefore, giving r a fixed small value is likely to be not restrictive.

In this section, we describe an estimation method which is clearly different from the one in [10]. More precisely, we generalize the Expectation-Maximization algorithm from [5], developed there for independent and identically distributed random variables, to a VAR process. For this purpose, we work with the full model (4) that includes the ISDM part pertaining to the r latent variables. Without loss of generality, we assume that $\Upsilon_{\ell }\left(\omega \right)$ equals the identity matrix I; the effect of the latent variables on the manifest ones in (5) can be modeled by $\Upsilon_{\ell m}$ alone. Combining with (2), the model is

(15) $$\Phi^{-1}\left(\omega \right)=\left[\begin{array}{cc} \Upsilon_{m}\left(\omega \right) & \Upsilon_{\ell m}^{⊤}\left(-\omega \right)\\ \Upsilon_{\ell m}\left(\omega \right) & I \end{array}\right]=\sum_{i=-p}^{p}Q_{i}e^{-j\omega i},$$

where the matrices $Q_{i}$ have to be found.

The main difficulty of this approach is the unavailability of the latent part of the matrices (11). Were such matrices available, we could work with SDM Φ(ω) estimators (confined to order *p*) of the form

(16) $$\tilde{\Phi }\left(\omega \right)=\sum_{i=-p}^{p}C_{i}e^{-j\omega i},$$

where $C_{i}$ denotes the *i*-th covariance lag for the VAR process $\left\{x_{t}\right\}$ (see also (1) and (11)). We split the matrix coefficients from (15) and (16) according to the size of manifest and latent variables, e.g.,

(17) $$C_{i}=\left[\begin{array}{cc} C_{m,i} & C_{\ell m,-i}^{⊤}\\ C_{\ell m,i} & C_{\ell ,i} \end{array}\right].$$

To overcome the difficulty, the Expectation-Maximization algorithm alternatively keeps fixed either the model parameters $Q_{i}$ or the matrices $C_{i}$, estimating or optimizing the remaining unknowns. The expectation step of Expectation-Maximization assumes that the ISDM $\Phi^{-1}\left(\omega \right)$ from (15) is completely known. Standard matrix identities [5] can be easily extended to matrix trigonometric polynomials for writing down the formula

(18) $$\begin{array}{c} \Phi \left(\omega \right)=\left[\begin{array}{cc} \Phi_{m}\left(\omega \right) & -\Phi_{m}\left(\omega \right)\Upsilon_{\ell m}^{⊤}\left(-\omega \right)\\ -\Upsilon_{\ell m}\left(\omega \right)\Phi_{m}\left(\omega \right) & I+\Upsilon_{\ell m}\left(\omega \right)\Phi_{m}\left(\omega \right)\Upsilon_{\ell m}^{⊤}\left(-\omega \right) \end{array}\right]. \end{array}$$

Identifying (16) with (18) gives expressions for estimating the matrices $C_{i}$, depending on the matrices $Q_{i}$ from (15). The upper left corner of (18) needs no special computation, since the natural estimator is

$$\Phi_{m}\left(\omega \right)=\sum_{i=-p}^{p}{\hat{R}}_{i}e^{-j\omega i},$$

where the sample covariances ${\hat{R}}_{i}$ are directly computable from the time series. It results that

(19) $$C_{m,i}={\hat{R}}_{i},\;\;i=\bar{-p,p}.$$

The other blocks from (17) result from convolution expressions associated with the polynomial multiplications from (18). The lower left block of the coefficients is

(20) $$C_{\ell m,i}=-\sum_{k+s=i}Q_{\ell m,k}{\hat{R}}_{s}=-\sum_{k=\max(-p,i-p)}^{\min(p,i+p)}Q_{\ell m,k}{\hat{R}}_{i-k},\;\;i=\bar{-2p,2p}.$$

Note that the trigonometric polynomial $\Upsilon_{\ell m}\left(\omega \right)\Phi_{m}\left(\omega \right)$ has degree 2p, since its factors have degree *p*. With (20) available, we can compute

(21) $$C_{\ell ,i}=\delta_{i}I+\sum_{k-s=i}C_{\ell m,k}Q_{\ell m,-s}^{⊤}=\delta_{i}I+\sum_{k=\max(-2p,i-p)}^{\min(2p,i+p)}C_{\ell m,k}Q_{\ell m,i-k}^{⊤},\;\;i=\bar{-p,p},$$

where $\delta_{i}=1$ if i=0 and $\delta_{i}=0$ otherwise. Although the degree of the polynomial from the lower right block of (18) is 3p, we need to truncate it to degree *p*, since this is the degree of the ISDM $\Phi^{-1}\left(\omega \right)$ from (15). This is the reason for computing only the coefficients $i=\bar{-p,p}$ in (21). The same truncation is applied on (20); note that there we cannot compute only the coefficients that are finally needed, since all of them are required in (21).

In the maximization step of Expectation-Maximization, the covariance matrices $C_{i}$ are assumed to be known and are fixed; the ISDM can be estimated by solving an optimization problem that will be detailed below. The overall solution we propose is outlined in Algorithm 1, explained in what follows.

**Algorithm 1** Algorithm for Identifying SP of ISDM (AlgoEM)
**Input:** $\mathrm{Data}\left(x_{1:K,1},\ldots ,x_{1:K,T}\right)$, VAR-order *p*, number of latent variables r, an information theoretic criterion (ITC).**Initialization:**Evaluate ${\hat{R}}_{i}$ for $i=\bar{0,p}$; (see (11) and the discussion below it)$\hat{R}\leftarrow \left[{\hat{R}}_{0}\ldots {\hat{R}}_{p}\right]$;${\hat{\Phi }}_{m}\left(\omega \right)\leftarrow \sum_{i=-p}^{p}{\hat{R}}_{i}e^{-j\omega i}$;${\left\{{\overset{ˇ}{Q}}_{i}^{\left(0\right)}\left(1:K,1:K\right)\right\}}_{i=0}^{p}\leftarrow {\mathrm{ME}}_{I}\left(\hat{R}\right)$ (see (22));Compute ${\overset{ˇ}{\Upsilon }}_{\ell m}^{\left(0\right)}\left(\omega \right)$ from EIG of ${\overset{ˇ}{Q}}_{0}^{\left(0\right)}$;**for all** $\lambda \in \{\lambda_{1},\ldots ,\lambda_{L}\}$ **do** **Maximum Entropy Expectation-Maximization (penalized setting):** **for** $\mathrm{it}=1,\ldots ,N_{\mathrm{it}}$ **do**  Use ${\hat{\Phi }}_{m}\left(\omega \right)$ and ${\overset{ˇ}{\Upsilon }}_{\ell m}^{\left(\mathrm{it}-1\right)}\left(\omega \right)$ to compute ${\overset{ˇ}{C}}^{\left(\mathrm{it}\right)}$ (see (16)–(18));  ${\left\{{\overset{ˇ}{Q}}_{i}^{\left(\mathrm{it}\right)}\right\}}_{i=0}^{p}\leftarrow {\mathrm{ME}}_{\mathrm{II}}\left({\overset{ˇ}{C}}^{\left(\mathrm{it}\right)},\lambda \right)$ (see (23));  Get ${\overset{ˇ}{\Upsilon }}_{\ell m}^{\left(\mathrm{it}\right)}\left(\omega \right)$ from ${\left\{{\overset{ˇ}{Q}}_{i}^{\left(\mathrm{it}\right)}\right\}}_{i=0}^{p}$ (see (15)); **end for** Use ${\left\{{\overset{ˇ}{Q}}_{i}^{\left(N_{\mathrm{it}}\right)}\right\}}_{i=0}^{p}$ to compute ${\overset{ˇ}{\Phi }}_{\lambda }^{-1}\left(\omega \right)$; Determine ${\mathrm{SP}}_{\lambda }$ (see (24)); **if** ADAPTIVE **then**  ${\overset{ˇ}{\Upsilon }}_{\ell m}^{\left(0\right)}\left(\omega \right)\leftarrow {\overset{ˇ}{\Upsilon }}_{\ell m}^{\left(N_{\mathrm{it}}\right)}\left(\omega \right)$
 **end if** ${\hat{\Upsilon }}_{\ell m}^{\left(0\right)}\left(\omega \right)\leftarrow {\overset{ˇ}{\Upsilon }}_{\ell m}^{\left(N_{\mathrm{it}}\right)}\left(\omega \right)$; **Maximum Entropy Expectation-Maximization (constrained setting):** **for** $\mathrm{it}=1,\ldots ,N_{\mathrm{it}}$ **do**  Use ${\hat{\Phi }}_{m}\left(\omega \right)$ and ${\hat{\Upsilon }}_{\ell m}^{\left(\mathrm{it}-1\right)}\left(\omega \right)$ to compute ${\hat{C}}^{\left(\mathrm{it}\right)}$ (see (16)–(18));  ${\left\{{\hat{Q}}_{i}^{\left(\mathrm{it}\right)}\right\}}_{i=0}^{p}\leftarrow {\mathrm{ME}}_{\mathrm{III}}\left({\hat{C}}^{\left(\mathrm{it}\right)},{\mathrm{SP}}_{\lambda }\right)$ (see (25));  Get ${\hat{\Upsilon }}_{\ell m}^{\left(\mathrm{it}\right)}\left(\omega \right)$ from ${\left\{{\hat{Q}}_{i}^{\left(\mathrm{it}\right)}\right\}}_{i=0}^{p}$ (see (15)); **end for** Use ${\left\{{\hat{Q}}_{i}^{\left(N_{\mathrm{it}}\right)}\right\}}_{i=0}^{p}$ to compute ${\hat{\Phi }}_{\lambda }^{-1}\left(\omega \right)$; Find the matrix coefficients of the VAR-model by spectral factorization of ${\hat{\Phi }}_{\lambda }^{-1}\left(\omega \right)$ and compute $\mathrm{ITC}(\mathrm{Data};{\mathrm{SP}}_{\lambda })$.**end for**$\hat{\mathrm{SP}}\leftarrow \arg\min_{\lambda }\mathrm{ITC}\left(\mathrm{Data};{\mathrm{SP}}_{\lambda }\right)$;


The initialization stage provides a first estimate for the ISDM, from which the Expectation-Maximization alternations can begin. An estimate for the left upper corner of $\Phi^{-1}\left(\omega \right)$ is obtained by solving the *classical* Maximum Entropy problem for a VAR(p)-model, using the sample covariances of the manifest variables. We present below the matrix formulation of this problem, which allows an easy implementation in CVX (Matlab-based modeling system for convex optimization) [14]. The mathematical derivation of the matrix formulation from the information theoretic formulation can be found in [6,9].

First Maximum Entropy Problem [${\mathrm{ME}}_{I}\left(\hat{R}\right)$]:

(22) $$\begin{array}{c} \begin{array}{cc} \min_{X} & \mathrm{tr}\left(T\left(\hat{R}\right)X\right)-\log\det X_{00}\\ s.t. & X⪰0 \end{array} \end{array}$$

The block Toeplitz operator T is defined in (12). The size of the positive semidefinite matrix variable X is K(p+1)×K(p+1). For all $i=\bar{0,p}$, the estimate ${\overset{ˇ}{Q}}_{i}^{\left(0\right)}\left(1:K,1:K\right)$ of the ISDM (15) is given by $D_{i}\left(X\right)$.

In order to compute an initial value for $\Upsilon_{\ell m}\left(\omega \right)$, we resort to the eigenvalue decomposition (EIG) of ${\overset{ˇ}{Q}}_{0}^{\left(0\right)}\left(1:K,1:K\right)$. More precisely, after arranging the eigenvalues of ${\overset{ˇ}{Q}}_{0}^{\left(0\right)}\left(1:K,1:K\right)$ in the decreasing order of their magnitudes, we have ${\overset{ˇ}{Q}}_{0}^{\left(0\right)}\left(1:K,1:K\right)=UDU^{⊤}$. Then, we set ${\overset{ˇ}{Q}}_{0}^{\left(0\right)}\left(K+1:K+r,1:K\right)=D^{1/2}\left(1:r,1:r\right)U^{⊤}\left(1:K,1:r\right)$ and ${\overset{ˇ}{Q}}_{i}^{\left(0\right)}\left(K+1:K+r,1:K\right)=0$ for $i=\bar{1,p}$.

When the covariances $C_{i}$ are fixed in the maximization step of the Expectation-Maximization algorithm, the coefficients of the matrix polynomial that is the ISDM (15) are estimated from the solution of the following optimization problem:

Second Maximum Entropy Problem [${\mathrm{ME}}_{\mathrm{II}}\left(C,\lambda \right)$]:

(23) $$\begin{array}{c} \begin{array}{cc} \min_{X} & \mathrm{tr}\left(T\left(C\right)X\right)-\log\det X_{00}+\lambda f\left(X\right)\\ s.t. & X⪰0\\ & D_{0}\left(X\right)\left(K+1:K+r,K+1:K+r\right)=I\\ & D_{i}\left(X\right)\left(K+1:K+r,K+1:K+r\right)=0,\;i=\bar{1,p} \end{array} \end{array}$$

Since now we work with the full model, the size of X is (K+r)(p+1)×(K+r)(p+1). The function f(·) is the sparsity promoter defined in (14) and depends only on the entries of the block corresponding to the manifest variables. The equality constraints in (23) guarantee that the latent variables have variance one and they are independent, given the manifest variables, corresponding to the lower right block of (15).

The estimates ${\left\{{\overset{ˇ}{Q}}_{i}^{\left(N_{\mathrm{it}}\right)}\right\}}_{i=0}^{p}$ obtained after these iterations are further employed to compute ${\overset{ˇ}{\Phi }}_{\lambda }^{-1}\left(\omega \right)$ by using (15). If λ is large enough, then ${\overset{ˇ}{\Phi }}_{\lambda }^{-1}\left(\omega \right)$ is expected to have a certain sparsity pattern, ${\mathrm{SP}}_{\lambda }$. Since the objective of (23) does not ensure exact sparsification and also because of the numerical calculations, the entries of ${\overset{ˇ}{\Phi }}_{\lambda }^{-1}\left(\omega \right)$ that belong to ${\mathrm{SP}}_{\lambda }$ are small, but not exactly zero. In order to turn them to zero, we apply a method similar to the one from [6] (Section 4.1.3). We firstly compute the maximum of partial spectral coherence (PSC),

(24) $$\begin{array}{c} \max_{\omega \in (-\pi ,\pi ]}\frac{\left|{\left[{\overset{ˇ}{\Phi }}_{\lambda }^{-1}\left(\omega \right)\right]}_{a\;b}\right|}{\sqrt{{\left[{\overset{ˇ}{\Phi }}_{\lambda }^{-1}\left(\omega \right)\right]}_{a\;a}{\left[{\overset{ˇ}{\Phi }}_{\lambda }^{-1}\left(\omega \right)\right]}_{b\;b}}}, \end{array}$$

for all a≠b with 1≤a,b≤K. Then ${\mathrm{SP}}_{\lambda }$ comprises all the pairs (a,b) for which the maximum PSC is not larger than a threshold Th. The discussion on the selection of parameters $N_{\mathrm{it}}$ and Th is deferred to Section 5.

The regularized estimate of ISDM is further improved by solving a problem similar to (23), but with the additional constraint that the sparsity pattern of ISDM is ${\mathrm{SP}}_{\lambda }$, more precisely:

Third Maximum Entropy Problem [${\mathrm{ME}}_{\mathrm{III}}\left(C,\mathrm{SP}\right)$]:

(25) $$\begin{array}{c} \begin{array}{cc} \min_{X} & \mathrm{tr}\left(T\left(C\right)X\right)-\log\det X_{00}\\ s.t. & X⪰0\\ & D_{0}\left(X\right)\left(K+1:K+r,K+1:K+r\right)=I\\ & D_{i}\left(X\right)\left(K+1:K+r,K+1:K+r\right)=0,\;i=\bar{1,p}\\ & D_{i}\left(X\right)\left(a,b\right)=0\;,i=\bar{0,p},\;\mathrm{if}\;\mathrm{SP}\left(a,b\right)=0 \end{array} \end{array}$$

This step of the algorithm has a strong theoretical justification which stems from the fact that ${\hat{\Phi }}^{-1}\left(\omega \right)$ is the Maximum Entropy solution for a covariance extension problem (see [10] (Remark 2.1)). The number of iterations, $N_{\mathrm{it}}$, is the same as in the case of the first loop.

The spectral factorization of the positive matrix trigonometric polynomial ${\hat{\Phi }}_{\lambda }^{-1}\left(\omega \right)$ is computed by solving a semidefinite programming problem. The implementation is the same as in [8], except that in our case the model contains latent variables. Therefore, the matrix coefficients produced by spectral factorization are altered to keep only those entries that correspond to manifest variables. The resulting VAR model is fitted to the data and then various IT criteria are evaluated. The accuracy of the selected model depends on the criterion that is employed as well as on the strategy used for generating the λ-values that yield the competing models. In the next section, we list the model selection rules that we apply; the problem of generating the λ-values is treated in Section 5.

As already mentioned, the estimation problem is solved for several values of λ: $\lambda_{1}<\lambda_{2}<\cdots <\lambda_{L}$. From the description above we know that, for each value of the parameter λ, $\Upsilon_{\ell m}\left(\omega \right)$ gets the same initialization, which is based on (22). It is likely that this initialization is poor. A better approach is an *ADAPTIVE* algorithm which takes into consideration the fact that the difference $\lambda_{i}-\lambda_{i-1}$ is small for all $i=\bar{2,L}$. This algorithm initializes $\Upsilon_{\ell m}\left(\omega \right)$ as explained above only when $\lambda =\lambda_{1}$. When $\lambda =\lambda_{i}$ for $i=\bar{2,L}$, the initial value of $\Upsilon_{\ell m}\left(\omega \right)$ is taken to be the estimate of this quantity that was previously obtained by solving the optimization problem in (23) for $\lambda =\lambda_{i-1}$. The effect of the *ADAPTIVE* procedure will be investigated empirically in Section 5.

The newly proposed estimation method outlined in Algorithm 1 is dubbed AlgoEM. The Matlab code for AlgoEM can be downloaded from https://www.stat.auckland.ac.nz/~cgiu216/PUBLICATIONS.htm.

## 4. Model Selection

### 4.1. IT Criteria

It is well-known that the IT criteria can be expressed as the sum of a goodness-of-fit (GOF) term and a penalty. They are derived on various grounds, but their expressions cannot be obtained easily for the problem we investigate. Due to this reason, we resort to the methodology applied previously to VAR without latent variables, where the criteria originally proposed for model order selection have been modified such that to be employed for finding the best sparsity pattern. As the GOF term is obtained straightforwardly by fitting the model to the data, the difficult part is the alteration of the penalty term. Based on the observation that all the penalty terms of the criteria for model order selection involve the number of parameters of the model, reference [6] proposed to replace it with the *effective number of parameters*:

(26) $$\begin{array}{c} N_{\mathrm{ef}}=\frac{K(K+1)}{2}-N_{0}+p\left(K^{2}-2N_{0}\right). \end{array}$$

Note that $N_{0}$ is the number of zeros in the lower triangular part of ${\mathrm{SP}}_{\lambda }$. The expression above can be obtained straightforwardly by counting the number of non-zero entries in the K×K upper-left block of the matrices ${\left\{{\hat{Q}}_{i}^{\left(N_{\mathrm{it}}\right)}\right\}}_{i=0}^{p}$ produced by Algorithm 1; counting takes into consideration all the existing symmetries.

The formula in (26) was used in [6] in order to modify three celebrated criteria: Schwarz Bayesian Criterion—SBC [15], Akaike Information Criterion—AIC [16] and its corrected version—${\mathrm{AIC}}_{c}$ [13] (pp. 432) and [17]. We name SBC the criterion from [15] because this is the term used in time series literature; the same selection rule is called Bayesian Information Criterion (BIC) in other works. Based on the empirical evidence from [6], SBC is ranked best when the sample size is large, whereas ${\mathrm{AIC}}_{c}$ works better for small sample sizes. This makes us to employ these two criteria in our experiments. Their expressions are [6]:

(27) $$\begin{array}{ccc} \mathrm{SBC}(\mathrm{Data};\mathrm{SP}) & = & T\log\det\hat{\Sigma }+N_{\mathrm{ef}}\log T,\\ {\mathrm{AIC}}_{c}\left(\mathrm{Data};\mathrm{SP}\right) & = & T\log\det\hat{\Sigma }+\frac{2N_{\mathrm{ef}}T}{T-N_{\mathrm{ef}}-1}, \end{array}$$

where $\hat{\Sigma }$ is the error covariance matrix. For simplicity, we write SP instead of ${\mathrm{SP}}_{\lambda }$. Even if our notation does not emphasize on this fact, it is clear that both $N_{\mathrm{ef}}$ and $\hat{\Sigma }$ depend on λ.

Another criterion employed in our tests is a variant of the Final Prediction Error—FPE [18]. The formula we use was obtained in [8] by relying on the asymptotic equivalence between logFPE and AIC. For ease of comparison with other criteria, we do not give the expression of FPE, but that of logFPE:

$$\begin{array}{ccc} \log\mathrm{FPE}(\mathrm{Data};\mathrm{SP}) & = & \log\det\hat{\Sigma }+K\log\frac{T+\eta }{T-\eta }, \end{array}$$

where $\eta =N_{\mathrm{ef}}/K$.

We also apply the Renormalized Maximum Likelihood (RNML) criterion. Its derivation is related to the problem of encoding losslessly measurements assumed to be a sequence of outcomes from an unknown distribution. It has been proven in [19] that there is a unique distribution which, if it is used to encode these measurements, then it leads to the minimum code length in the worst case scenario. This particular distribution was further utilized in [20,21] in order to introduce the RNML criterion for model selection. The major problem comes from the fact that, for most of the family of models, its closed-form expression is hard to be obtained. The expression of RNML that can be employed for choosing the order of VAR-models was firstly derived in [22]. The properties of this criterion have been investigated in [8], where the criterion was also altered such that to be applied in the selection of the sparsity pattern for ISDM of VAR-models. With our notation, the RNML criterion can be written as follows:

(28) $$\mathrm{RNML}\left(\mathrm{Data};\mathrm{SP}\right)=\frac{T-\eta -K+1}{2}\log\det\hat{\Sigma }+\frac{N_{\mathrm{ef}}}{2}\log\mathrm{tr}\left({\hat{R}}_{0}-\hat{\Sigma }\right)-\log\Gamma_{K}\left(\frac{T-\eta }{2}\right)-\log\Gamma \left(\frac{N_{\mathrm{ef}}}{2}\right),$$

where $\Gamma_{K}\left(\cdot \right)$ is the multivariate Gamma function and Γ(·) is the Gamma function. The matrix ${\hat{R}}_{0}$ has the same significance as in (12). It is remarkable that the penalty term of RNML depends on the actual measurements, and not only on the triple $\left(T,K,N_{\mathrm{ef}}\right)$.

A common feature of all the criteria presented so far is that they do not take into consideration how big is the family of the competing candidates. This might be problematic because the total number of possible sparsity patterns is as large as $2^{\bar{K}}$, where $\bar{K}=K\left(K-1\right)/2$. The solutions proposed in the previous literature for circumventing this difficulty are called *extended* IT criteria. We show below how these criteria can be applied for selecting the sparsity pattern. In this context, we also propose a novel variant of RNML.

### 4.2. Extended IT Criteria

First we write down the expression of the extended SBC proposed in [23]. In the statistical literature, this criterion is named EBIC (Extended Bayesian Information Criterion):

(29) EBIC(Data;SP)=SBC(Data;SP)+2γlogK¯N0,

where γ∈[0,1]. It is evident that EBIC is equivalent to SBC when γ=0. More interestingly, reference [24] uses arguments from information theory for justifying the use of a penalty term which counts the number of models that have the same number of parameters. For our problem, this is equivalent to choosing γ=1 in (29). Because this is also the value of γ that we use in this work for evaluating EBIC, we explain briefly the significance of the supplementary penalty term. The key point is to consider a scenario in which Data should be transmitted losslessly from an encoder to a decoder by employing the model given by SP. According to [24], the first step is to transmit the value of $N_{0}$. The assumption that all possible values of $N_{0}$ are equally probable leads to the conclusion that the code length for $N_{0}$ is $-\log\left(1/(\bar{K}+1)\right)=\log\left(\bar{K}+1\right)$. As this quantity is the same for all models, it can be neglected. Then the decoder should be informed about the actual locations of the zeros in the sparsity pattern SP. Since the list of all sparsity patterns for a given $N_{0}$ is known by both the encoder and the decoder, all that remains is to send to the decoder the index of SP in this list. Under the hypothesis that all the sparsity patterns in the list are equally probable, the code length for the index is logK¯N0. In (29), this quantity is multiplied by two because of the scaling factor used in the definition of SBC(Data;SP).

Another formulation of EBIC was introduced in [25] for finding the graphical structure of a Gaussian model (static case), in the situation when the number of variables and the number of observations grow simultaneously. After modifying the criterion from [25] by replacing the number of edges of the graph with $N_{\mathrm{ef}}$, we get the following formula:

(30) $$\begin{array}{c} {\mathrm{EBIC}}_{\mathrm{FD}}\left(\mathrm{Data};\mathrm{SP}\right)=\mathrm{SBC}\left(\mathrm{Data};\mathrm{SP}\right)+4\gamma N_{\mathrm{ef}}\log K, \end{array}$$

where γ has the same significance as in (29), and again we take γ=1. Remark that the term $4\gamma N_{\mathrm{ef}}\log K$ grows when $N_{0}$ decreases; the term in (29), 2γlogK¯N0=2γlogK¯K¯−N0, does not have the same property.

Relying on the asymptotic equivalence between RNML and SBC [8], we alter RNML by adding half of the extra penalty from (30); the scaling factor is needed because the ratio between the GOF term in (28) and the GOF term in (27) tends to 1/2 when T→∞. The new criterion, which is dubbed ${\mathrm{RNML}}_{\mathrm{FD}}$, has the following expression:

(31) $$\begin{array}{c} {\mathrm{RNML}}_{\mathrm{FD}}\left(\mathrm{Data};\mathrm{SP}\right)=\mathrm{RNML}\left(\mathrm{Data};\mathrm{SP}\right)+2N_{\mathrm{ef}}\log K. \end{array}$$

For all the selection rules listed above, the best model is the one which minimizes the value of the criterion. For evaluating the performance of IT criteria, we conduct an empirical study. The main results of this study are reported in the next section.

## 5. Experimental Results

### 5.1. Artificial Data

**Figure 1:**
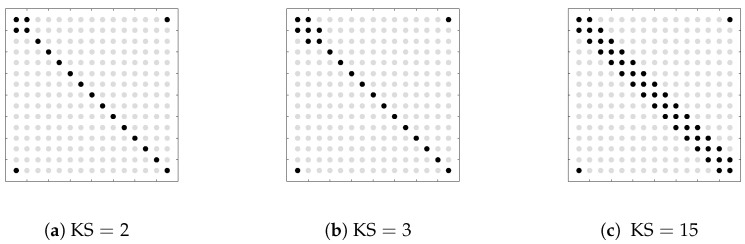
Sparsity patterns for the ISDM of the generated data: KS is the number of non-zero entries in the lower triangular part of SP. The black dots represent the locations of the non-zero entries, whereas the light grey dots are the zero entries.

In our simulations, the order of the VAR model in (1) is taken to be one, the number of manifest variables is K=15, and there is one single latent variable (r=1). Let KS denote the number of non-zero entries in the lower triangular part of the sparsity pattern of size K×K. We consider three different values for KS: 2, 3 and K. Remark that the larger is KS, less sparse is the ISDM. The locations of the non-zero entries for each value of KS are graphically represented in Figure 1.

When generating the ISDM, all the matrices ${\left\{Q_{i}\right\}}_{i=0}^{p}$ in (2) have only ones on their main diagonals. The entries of the K×K upper-left block of the matrix $Q_{i}$, which should be non-zero according to Figure 1, are equal to 0.5/(i+1). Additionally, the entries on the last row and on the last column of $Q_{i}$, except the one on the main diagonal, are equal to 0.3/(i+1). Integer multiples of the κ×κ identity matrix are added to $Q_{0}$ until the resulting ISDM is positive definite. Furthermore, the spectral factorization is applied in order to obtain the matrix coefficients of the VAR-model from the ISDM (see [8] (Section 4.2) for more details). Hence, for each value of KS, one single VAR-model is produced and this is used for generating $N_{\mathrm{tr}}=10$ κ-variate time series of length T= 50,000. To this end, we utilize Matlab R2014b functions from the package available at the address http://climate-dynamics.org/software/#arfit. After discarding from each time series the component corresponding to the latent variable, the simulated data are used for evaluating the performance of AlgoEM.

### 5.2. Settings for AlgoEM

The order of VAR, as well as the number of latent variables, is assumed to be known. The parameter λ takes values on a regular grid defined on the interval $\left[10^{-3},10^{-1}\right]$, for which the grid step is $10^{-3}$. It follows that the total number of values for λ is L=100. The threshold Th, which is used in conjunction with (24) in order to get the estimated sparsity pattern, equals $10^{-3}$.

**Figure 2:**
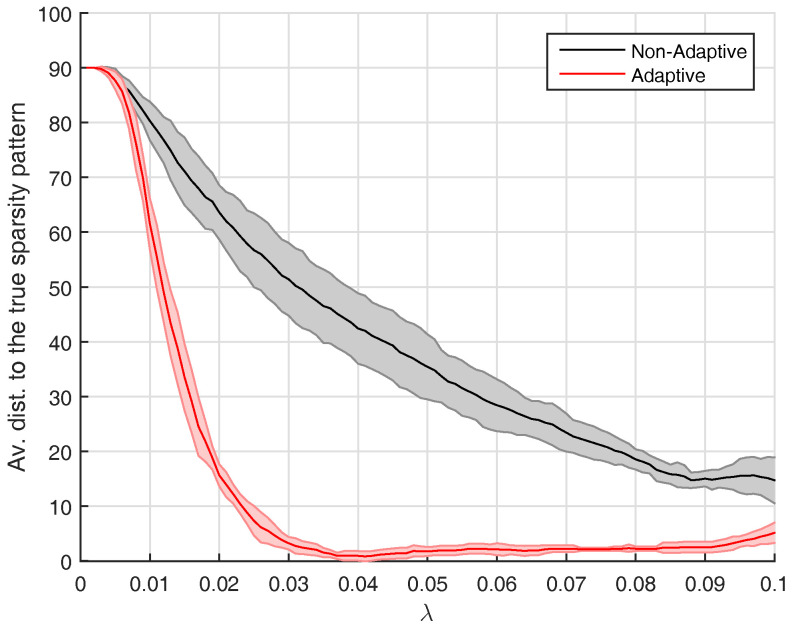
Results for VAR-models with KS=15, for which the true sparsity pattern is shown in Figure 1c. With the convention that dist denotes the distance between the estimated sparsity pattern and the true one, we plot mean(dist) ±1 standard deviation(dist) versus the parameter λ. The statistics are computed from $N_{\mathrm{tr}}=10$ trials, for both the *adaptive* and the *non-adaptive* case.

We are interested to evaluate the impact of the adaptive initialization procedure that was introduced in Section 3. This is why we run AlgoEM with and without this procedure, for all the time series we have generated. For each time series and for each value of λ on the grid, the estimated ${\mathrm{SP}}_{\lambda }$ is compared to the true sparsity pattern. The comparison reduces to computing the distance between the two sparsity patterns, which is given by the number of positions below the main diagonal where the patterns differ. For the case KS=15, statistics related to this distance are presented in Figure 2. Remark that values of λ close to zero lead to estimated patterns which are not sparse. As expected, this happens disregarding if the adaptive procedure is applied or not. The use of the procedure has the positive effect that, for a large range of λ-values, the estimated patterns are close to the true one. The same is true for both KS=2 and KS=3, which makes us apply the adaptive procedure in all the experiments outlined below.

**Figure 3:**
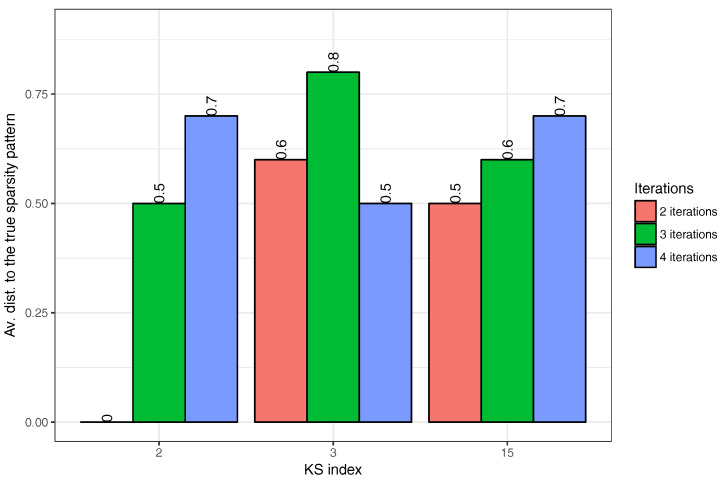
Impact of $N_{\mathrm{it}}$ on the performance of AlgoEM: Evaluation is done by replacing in AlgoEM the IT criterion with an oracle having full knowledge about the true sparsity pattern. For each KS and for each $N_{\mathrm{it}}$, we run $N_{\mathrm{tr}}=10$ trials for calculating the average distance between the true sparsity pattern and the sparsity pattern selected by oracle.

The results reported in Figure 2 are obtained by taking the number of iterations to be $N_{\mathrm{it}}=4$. As the computational burden of the algorithm depends strongly on $N_{\mathrm{it}}$, we investigate the effect of reducing the number of iterations to $N_{\mathrm{it}}=3$ and $N_{\mathrm{it}}=2$, respectively. In each case, the evaluation of performance is done by an oracle having complete knowledge about the true sparsity pattern. From the set of sparsity patterns produced when applying AlgoEM to a particular time series, the oracle selects the one which is closest to the true sparsity pattern. The closeness is measured by the distance defined above. The average distances computed from $N_{\mathrm{tr}}=10$ trials are plotted in Figure 3. We can see in the figure that, in the case when KS=2 and AlgoEM performs only two iterations, the true sparsity pattern is always in the set of the candidates produced by the algorithm and this makes the average distance to be zero. In general, all the results shown in the figure are good as the average distance is smaller than one in all cases. Since the increase of $N_{\mathrm{it}}$ does not guarantee the improvement in performance, we take $N_{\mathrm{it}}=2$ for reducing the complexity of the algorithm.

**Figure 4:**
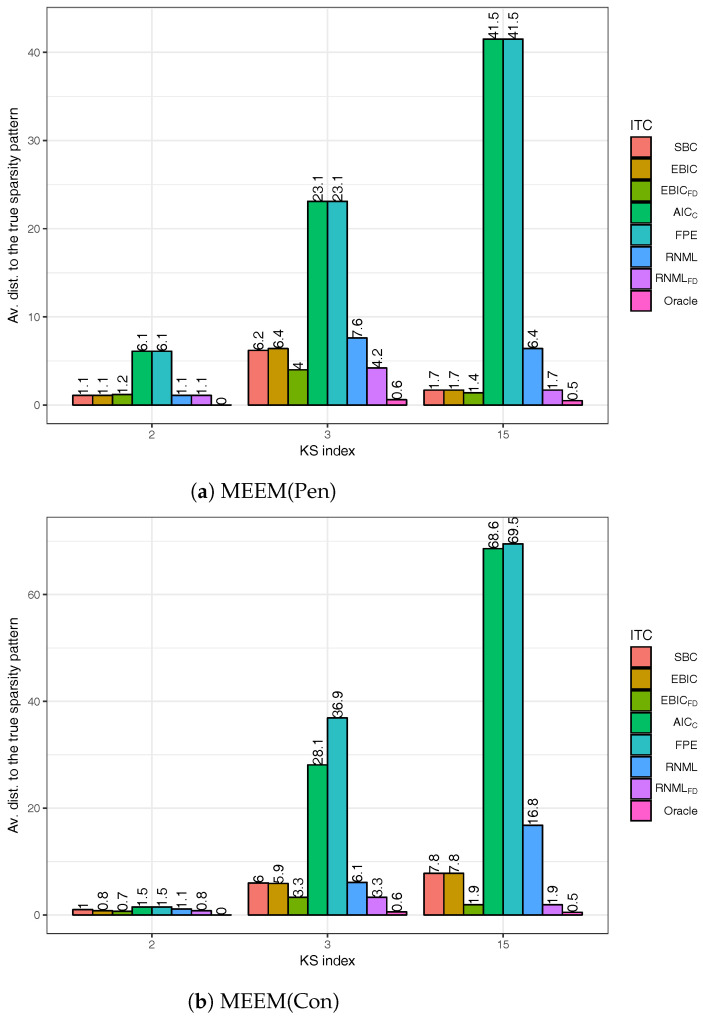
Performance of IT criteria compared to that of an oracle: (**a**) Only the first major loop, MEEM(Pen), of AlgoEM is executed; (**b**) Both MEEM(Pen) and MEEM(Con) are executed.

It is clear from the description of Algorithm 1 that $N_{\mathrm{it}}$ is the same for the two major loops of AlgoEM. We name the first loop MEEM(Pen) and the second one MEEM(Con). Obviously, MEEM is the acronym for Maximum Entropy Expectation-Maximization, Pen stands for *penalized* settings, and Con means *constrained* settings. Because we want to quantify the influence of MEEM(Con) on the accuracy of the estimation, we show in Figure 4 the average distances to the true pattern, when the estimates are produced by MEEM(Pen) and by MEEM(Con), respectively. This time we do not report results obtained only when an oracle is used for selecting the sparsity pattern, but also for the case when the selection is done with the IT criteria defined in Section 4. We can observe that ${\mathrm{AIC}}_{c}$ and FPE perform very poorly, whereas both ${\mathrm{EBIC}}_{\mathrm{FD}}$ and ${\mathrm{RNML}}_{\mathrm{FD}}$ are very good. The fact that ${\mathrm{RNML}}_{\mathrm{FD}}$ is superior to RNML demonstrates the importance of the extra-term in (31). Remark also that ${\mathrm{EBIC}}_{\mathrm{FD}}$ is more accurate than SBC, while EBIC has the same level of performance as SBC. The most important conclusion is that removing MEEM(Con) from AlgoEM does not deteriorate the final outcome.

The next step is to compare AlgoEM with the algorithm that solves the optimization problem in (13). We use the implementation from [12], which is publicly available at the address https://drive.google.com/file/d/0BykD2O6uX6KjSGlkQTRYWFVBZGM/view, and we call it AlgoSL. The name comes from the fact that the method in (13) is *sparse plus low-rank*.

### 5.3. Comparison of AlgoEM and AlgoSL

In [12], the sparsity pattern for a given pair of parameters (λ,γ) is found by solving (13). Reference [10] uses further this sparsity pattern as an initialization for a constrained optimization problem (see also the discussion below Equation (25)). In both cases, a set of sparsity patterns is generated by choosing various values for the parameters λ and γ. For selecting the best one, they consider an alternative to IT criteria, which is dubbed *score function* (SF). The key point is that, when using SF, it is not needed to compute explicitly the matrix coefficients of the VAR-model. More details are provided below.

Let ${\hat{\Phi }}_{m}$ be an estimate of $\Phi_{m}$ in (4), which is constrained to have a certain sparsity pattern, SP. Using an idea from [9], reference [10] suggests to employ Data for computing the correlogram ${\hat{\Phi }}_{m}^{c}$ (with Bartlett window) [26], and then to evaluate the relative entropy rate:

(32) $$\begin{array}{c} D({\hat{\Phi }}_{m}^{c}\left|\right|{\hat{\Phi }}_{m})=-\frac{1}{4\pi }\int_{-\pi }^{\pi }\log\det\Omega \left(\omega \right)+\mathrm{tr}\left(I-\Omega \left(\omega \right)\right)d\omega , \end{array}$$

where $\Omega \left(\omega \right)={\hat{\Phi }}_{m}^{c}\left(\omega \right){\hat{\Phi }}_{m}^{-1}\left(\omega \right)$ for all ω∈(−π,π]. This formula can be regarded as a generalization of the *I*-divergence of two positive definite matrices, which was originally introduced in connection with graphical models (static case) [3]. The expression in (32) was also employed by [27] in the inference of graphical models for time series. Some of the properties of the relative entropy rate are discussed in [28]. It is worth noting that this index also belongs to the Tau divergence family [29] and to the Beta divergence family [30]. It has been proven in these references that the use of (32) in the formulation of the Maximum Entropy problem leads to the simplest solution, in the sense of the minimum McMillan degree.

The most important is that, in [10], $D({\hat{\Phi }}_{m}^{c}||{\hat{\Phi }}_{m})$ is utilized for quantifying the adherence of the model to the data. The complexity of the model is determined by $N_{e}$, the total number of edges in the graph (including the edges that connect the latent variables to the manifest variables). For instance, if the model has a single latent variable, then $N_{e}=K\left(K+1\right)/2-N_{0}$. Note that $N_{0}$ has the same significance as in (26). It follows that $N_{\mathrm{ef}}=N_{e}+p\left(K^{2}-2N_{0}\right)$, which leads to the conclusion that $N_{\mathrm{ef}}>N_{e}$, and the difference between the two quantities increases when the order of the model raises. If p=1 and the number of latent variables is at least three (r≥3), then $N_{\mathrm{ef}}<N_{e}$ when $N_{0}$ is large.

The score functions given in [10] are:

(33) $$\begin{array}{ccc} \log{\mathrm{SF}}_{1}\left(\mathrm{Data};\mathrm{SP}\right) & = & \log D({\hat{\Phi }}_{m}^{c}\left|\right|{\hat{\Phi }}_{m})+\log N_{e}, \end{array}$$

(34) $$\begin{array}{ccc} {\mathrm{SF}}_{2}\left(\mathrm{Data};\mathrm{SP}\right) & = & D({\hat{\Phi }}_{m}^{c}\left|\right|{\hat{\Phi }}_{m})+\frac{N_{e}}{T}, \end{array}$$

(35) $$\begin{array}{ccc} {\mathrm{SF}}_{3}\left(\mathrm{Data};\mathrm{SP}\right) & = & D({\hat{\Phi }}_{m}^{c}\left|\right|{\hat{\Phi }}_{m})+\frac{N_{e}\log T}{T}, \end{array}$$

where D(·||·) is defined in (32). The formula of ${\mathrm{SF}}_{1}$ is logged for ease of reading.

**Figure 5:**
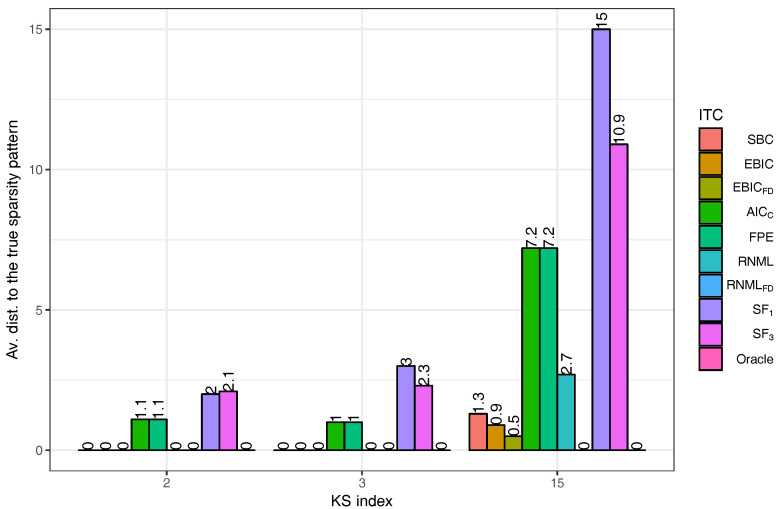
Estimation results obtained when AlgoSL is applied to the same time series which have been used to evaluate the performance of AlgoEM in Figure 4. For selection of the sparsity pattern, we employ the score functions in (33)–(35) and the IT criteria from Section 4. Score function ${\mathrm{SF}}_{2}$ is not shown in the graph because it leads to large values of the average distance: 102.4 for KS=2, 101.5 for KS=3, and 89.1 for KS=15.

We apply AlgoSL to all the time series we have simulated (see again Section 5.1). In our experiments, we consider the pairs (λ,γ) for which $\lambda \in \left\{0.1,0.2,\ldots ,0.6\right\}$ and $\gamma \in \left\{0.01,0.02,\ldots ,0.5\right\}$. For the selection of the sparsity pattern, we do not use only the score functions in (33)–(35), but we also employ the IT criteria from Section 4. The results are shown in Figure 5. The best criterion is ${\mathrm{RNML}}_{\mathrm{FD}}$, which is able to find the true sparsity pattern in all the experiments; the second best is ${\mathrm{EBIC}}_{\mathrm{FD}}$. The comparison of the plots in Figure 5 to those in Figure 4 leads to the conclusion that AlgoSL works better than AlgoEM when ${\mathrm{RNML}}_{\mathrm{FD}}$ is employed for selecting the model. For understanding these results, we should take into consideration two important aspects: (i) Oracle gives perfect results for all KS in the case of AlgoSL, but not in the case of AlgoEM; to some extent this is due to the fact that 300 (λ,γ)-pairs allow to produce a better set of candidates for AlgoSL than the one generated by 100 λ-values for AlgoEM; (ii) The score functions have been used in [10] for time series of hundreds of samples, whereas the size of the time series we simulated is much larger (T= 50,000).

**Figure 6:**
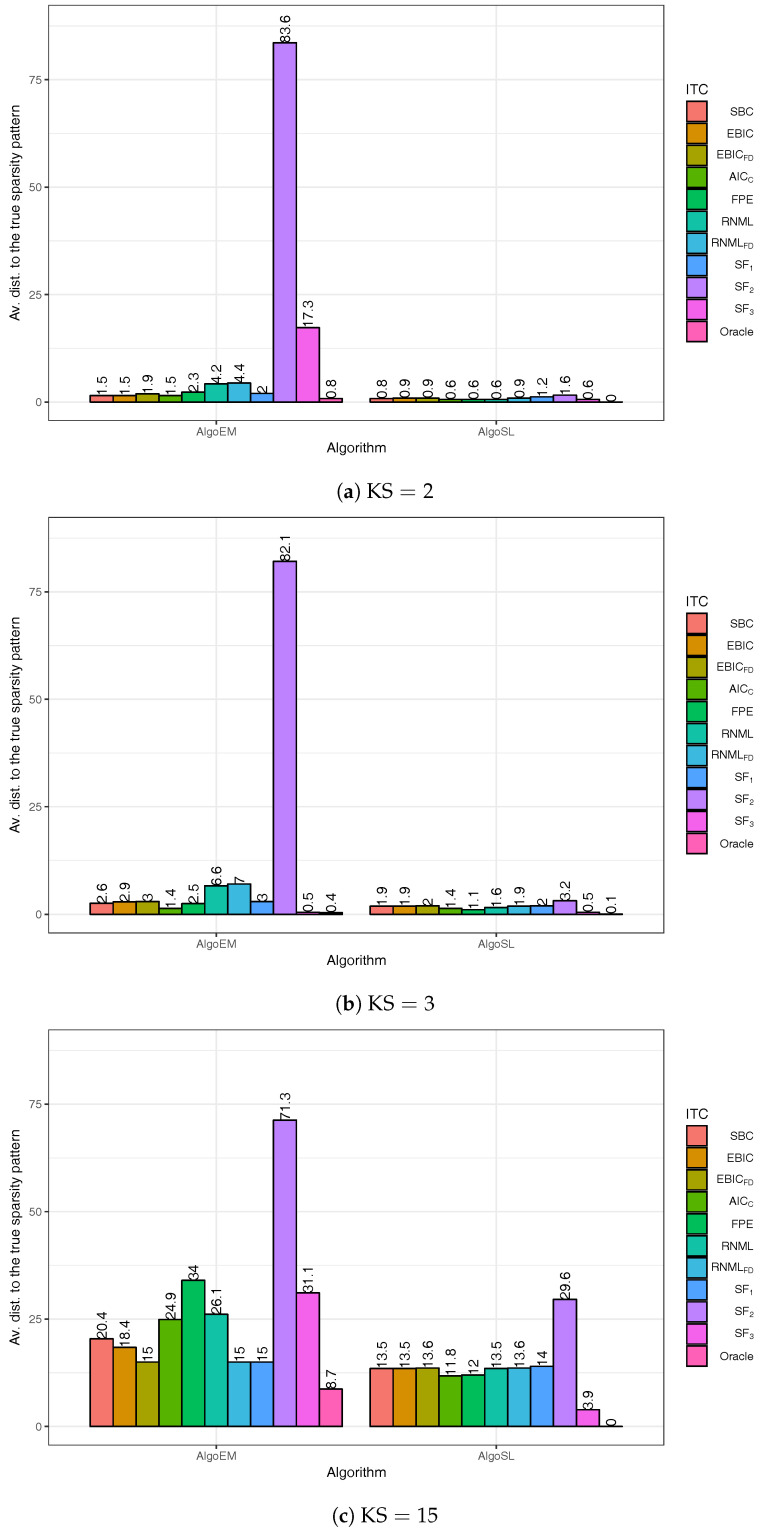
Estimation results when sample size is small (T=500).

In order to clarify the second aspect, we conduct an experiment with simulated time series for which T=500. The simulation procedure is the same as in Section 5.1 and all the settings are the same, except that the non-zero entries of the matrices ${\left\{Q_{i}\right\}}_{i=0}^{p}$ (which are not located on the main diagonal) have values that are fifty times larger. For AlgoEM, the uniform grid for λ takes values on the interval $\left[10^{-3},10^{-1}\right]$; the grid step is $10^{-3}$ (see also [31]). Based on some empirical evidence, we use the parameters $\lambda \in \left\{0.02,0.03,\ldots ,0.3\right\}$ and $\lambda \gamma \in \left\{0.01,0.015,\ldots ,0.2\right\}$ for AlgoSL. All IT criteria and all the score functions are used for both AlgoEM and AlgoSL, and the estimation results are reported in Figure 6. In general, they are worse than the results for large sample size (T= 50,000), and the difference is more evident when KS=15. This shows that, for small *T*, it might be difficult to recover the true structure of ISDM when it is not sparse enough. It is encouraging that, even for KS=15, oracle used in conjunction with AlgoSL finds the true pattern in all trials.

**Figure 7:**
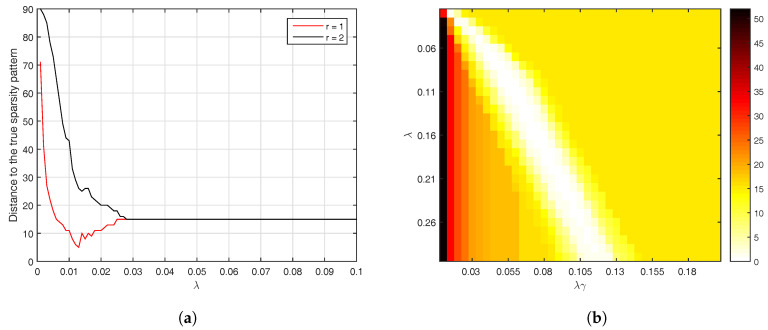
Distances computed for sparsity patterns which are estimated from a time series of length T=500; the value of KS for the true sparsity pattern is 15. (**a**) AlgoEM (*r* is the number of latent variables used in estimation); (**b**) AlgoSL.

To gain more insight, we give in Figure 7 a graphical representation of all the distances which are computed by oracle for a time series whose ISDM has KS=15. It follows from the way in which we have chosen the experimental parameters that the total number of such distances calculated for AlgoSL is more than eleven times larger than the number of distances for AlgoEM. We should keep in mind that we need to compute the distance from the pattern estimated by each candidate in the list to the true sparsity pattern. After a laborious process of selecting the values of λ and λγ, we ended-up with a two-dimensional grid which yields a good number of estimated patterns that are identical to the true pattern (see the white area inside the square shown in Figure 7b). The behavior of AlgoEM is different: For a large range of λ-values, the K×K upper-left block of the estimated pattern is equal to the identity matrix, which makes the distance to the true pattern to be equal to KS. This happens when the number of latent variables used in AlgoEM equals the true number (r=1) as well as when r=2 is utilized in estimation. However, it is evident in Figure 7a that the estimation results are better when r=1. If the number of latent variables is not known a priori, one possibility might be to use an ITC for selecting it. Note that the definition of $N_{\mathrm{ef}}$ in (26) should be modified. The score functions given in (33)–(35) do not need to be altered in order to be employed in selection of the number of latent variables.

### 5.4. Real-World Data

In addition to the experiments with simulated data, we test the capabilities of AlgoEM on multivariate time series of daily stock markets indices at closing time, which can be downloaded from the following address: http://au.mathworks.com/matlabcentral/fileexchange/48611-international-daily-stock-return-data-for-system-identification. This dataset was produced by pre-procesing the original data from http://finance.yahoo.com, which have been measured from 4 January 2012 to 31 December 2013. We refer to [10] for details regarding the pre-processing. The time series is K-variate with K=22, and the sample size is T=518.

For each component of the time series, we give the name of the country where was measured and we write in parentheses the acronym used in this analysis: Australia (AU), New Zealand (NZ), Singapore (SG), Hong Kong (HK), China (CH), Japan (JA), Korea (KO), Taiwan (TA), Brazil (BR), Mexico (ME), Argentina (AR), Switzerland (SW), Greece (GR), Belgium (BE), Austria (AS), Germany (GE), France (FR), Netherlands (NL), United Kingdom (UK), United States (US), Canada (CA), Malaysia (MA). The official names of the price indices can be found in [10].

**Figure 8:**
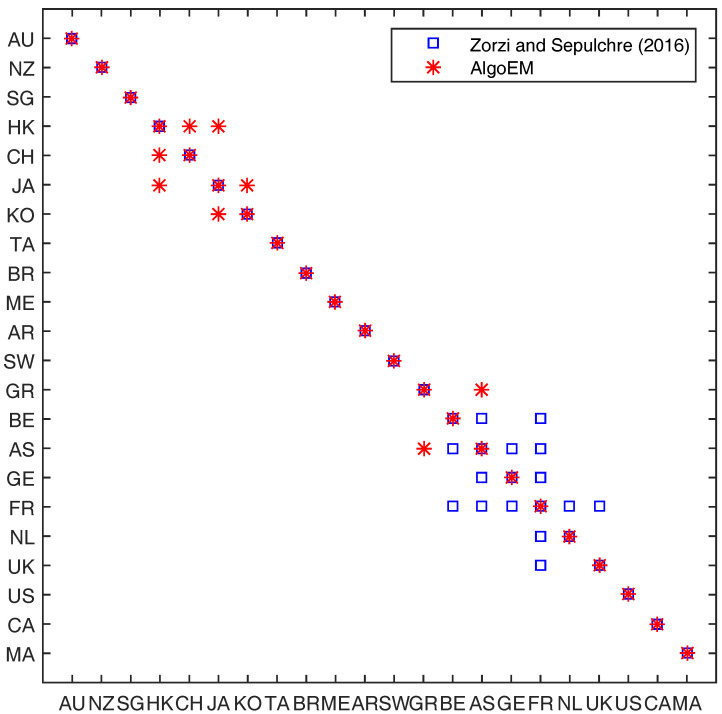
International stock markets data: Comparison between the sparsity pattern for manifest variables from [10] and the pattern produced by AlgoEM when either ${\mathrm{SF}}_{1}$ or one of the following IT criteria is used: SBC, EBIC, ${\mathrm{EBIC}}_{\mathrm{FD}}$, FPE, RNML, ${\mathrm{RNML}}_{\mathrm{FD}}$.

Similar to [10], we take the order of VAR-model to be p=1, and we assume that there is a single latent variable (r=1). For AlgoEM, we have $N_{\mathrm{it}}=4$ and λ takes values on a uniform grid on the interval $\left[2\times 10^{-3},2\times 10^{-1}\right]$, for which the grid step is $2\times 10^{-3}$. When ${\mathrm{AIC}}_{c}$ is used for choosing the model, the results are disappointing because the selected sparsity pattern contains very few zeros. The same type of outcome is also obtained when applying ${\mathrm{SF}}_{2}$ or ${\mathrm{SF}}_{3}$. It is interesting that ${\mathrm{SF}}_{1}$ as well as six different IT criteria (SBC, EBIC, ${\mathrm{EBIC}}_{\mathrm{FD}}$, FPE, RNML, ${\mathrm{RNML}}_{\mathrm{FD}}$) select exactly the same sparsity pattern. This one is compared in Figure 8 to the sparsity pattern given in [10], for the same data set. Observe that the conditional independence graph yielded by AlgoEM has only four edges which connect manifest variables: Three of them connect vertices corresponding to Asian markets, (HK,CH),(HK,JA),(JA,KO), and the fourth one connects two European markets, (GR,AT). This is different from the conditional graph in [10], where all the edges between manifest variables connect only the European markets (with the exception of Greece). An in-depth analysis of the two graphs is beyond the interest of this paper.

## 6. Final Remarks

The main motivation for this study is the solution proposed in [5] for the static case, as an alternative to the method from [4]. We have shown how the estimation method from [5] can be generalized for the dynamic case. The resulting algorithm is dubbed AlgoEM. We have conducted an empirical study in which we have investigated the capabilities of AlgoEM and we have also compared it with the generalization of the method from [4], which was proposed in [10]. It is important to emphasize that the two methods for latent-variable autoregressive models, which we have compared, have some common features: apply the Maximum Entropy principle, use convex optimization, and generate a set of candidate models from which the best one is selected by a certain rule. In the case of AlgoEM, the set of the candidates depends strongly on the parameter λ, which is chosen by the user. For the method in [10], the user should choose two parameters, λ and γ. Based on our experience, the selection of the two parameters is much more difficult than the selection of the single parameter for AlgoEM. Another important aspect is how to pick-up the winner from the competing models. In [10], this was restricted to the use of score functions. We have demonstrated empirically that the IT criteria might be an option to consider. Especially when the sample size is large, it is recommended to employ the criterion ${\mathrm{RNML}}_{\mathrm{FD}}$ which we have introduced in this work.
